# Differential analysis of immune reconstitution after allogeneic hematopoietic stem cell transplantation in children with Wiskott-Aldrich syndrome and chronic granulomatous disease

**DOI:** 10.3389/fimmu.2023.1202772

**Published:** 2023-06-14

**Authors:** Ya Zhou, Luying Zhang, Yan Meng, Xiaoying Lei, Lanzhou Jia, Xianmin Guan, Jie Yu, Ying Dou

**Affiliations:** Department of Hematology Oncology Children’s Hospital of Chongqing Medical University, National Clinical Research Center for Child Health and Disorders, Ministry of Education Key Laboratory of Child Development and Disorders, China International Science and Technology Cooperation base of Child development and Critical Disorders, Children’s Hospital of Chongqing Medical University, Chongqing Key Laboratory of Child Infection and Immunity, Children’s Hospital of Chongqing Medical University, Chongqing, China

**Keywords:** immune reconstruction, lymphocyte reconstruction, allogeneic hematopoietic stem cell transplantation, Wiskott-Aldrich syndrome, chronic granulomatous disease

## Abstract

**Objective:**

To investigate similarities and differences in immune reconstitution after allogeneic hematopoietic stem cell transplantation (allo-HSCT) in children with Wiskott-Aldrich syndrome (WAS) and chronic granulomatous disease (CGD).

**Method:**

We retrospectively analyzed the lymphocyte subpopulations and the serum level of various immune-related protein or peptide on Days 15, 30, 100, 180 and 360 post-transplantation in 70 children with WAS and 48 children with CGD who underwent allo-HSCT at the Transplantation Center of the Department of Hematology-Oncology, Children’s Hospital of Chongqing Medical University from January 2007 to December 2020, and we analyzed the differences in the immune reconstitution process between the two groups.

**Results:**

① The WAS group had higher lymphocyte subpopulation counts than the CGD group. ② Among children aged 1-3 years who underwent transplantation, the WAS group had higher lymphocyte subpopulation counts than the CGD group. ③ Further comparisons were performed between children with non-umbilical cord blood transplantation (non-UCBT) and children with umbilical cord blood transplantation (UCBT) in the WAS group. On Day 15 and 30 post-transplantation, the non-UCBT group had higher B-cell counts than the UCBT group. On the remaining time points post-transplantation, the UCBT group had higher lymphocyte subpopulation counts than the non-UCBT group. ④ Comparisons were performed between children with non-UCBT in the WAS group and in the CGD group, the lymphocyte subpopulation counts were higher in the WAS group compared to the CGD group. ⑤ On Day 100 post-transplantation, the CGD group had higher C3 levels than the WAS group. On Day 360 post-transplantation, the CGD group had higher IgA and C4 levels than the WAS group.

**Conclusion:**

① The rate of immunity recovery was faster in children within the WAS group compared to those children within the CGD group, which may be attributed to the difference of percentage undergoing UCBT and primary diseases. ② In the WAS group, the non-UCBT group had higher B-cell counts than the UCBT group at Day 15 and 30 post-transplantation, however, the UCBT group had higher B-cell counts than the non-UCBT group at Day 100 and 180 post-transplantation, suggesting that cord blood has strong B-cell reconstitution potentiality after transplantation.

## 1 Introduction

Allogeneic hematopoietic stem cell transplantation (allo-HSCT) is a treatment that transplants hematopoietic stem cells from a healthy donor into the patient and reestablishes hematopoiesis and immunity in the recipient through these stem cells with normal hematopoietic function. Currently, allo-HSCT can treat not only hematologic diseases but also certain immunodeficiency diseases and inherited metabolic disorders. The effectiveness of transplantation depends largely on the hematopoietic and immune reconstitution ability of the donor’s hematopoietic stem cells in the recipient, suggesting that post-transplantation hematopoietic and immune reconstitution is fundamental to the success of allo-HSCT. Delayed immune reconstitution may lead to an increased risk of infection-related death, which is a significant barrier to successful recovery from allo-HSCT (1).

Wiskott-Aldrich syndrome (WAS) and chronic granulomatous disease (CGD) are two different types of primary immunodeficiency diseases (PIDs). WAS, a syndromic combined immunodeficiency disease marked by an impaired lymphocyte lineage, is an X-linked invisible genetic disorder that usually affects males and is carried by females. WAS is prompted by mutations in WAS genes encoding the WAS protein (WASp), in which WASp is expressed only in the hematopoietic system and plays an important role in hematopoietic cell differentiation and migration, cell signaling, immune synapse formation, and lymphocyte apoptosis. As many as 300 mutations in WAS genes are known, resulting in varying degrees of defective WASp, and this can contribute to a variety of abnormal immune cell functions and lead to a variety of clinical phenotypes (2, 3). WAS features an increased incidence of congenital thrombocytopenia, eczema, recurrent infections, autoimmune diseases, and malignancies (4). Without radical treatment, most patients suffer from recurrent infections and long-term immunodeficiency, which affects the quality of life and life expectancy. CGD, an X-linked or autosomal recessive sickness, was brought on by mutations in the gene encoding a subunit of the NADPH oxidase complex, resulting in an inherited defect in phagocytosis (5). Patients with CGD are prone to recurrent bacterial and fungal infections, excessive inflammatory responses, and immune dysregulation due to defective oxygen radical production by phagocytes, which cannot effectively clear infections.

Currently, allo-HSCT offers the potential to cure PIDs such as WAS and CGD in the long term, along with the prophylactic use of antibacterial and antifungal drugs and improved symptomatic control of infections, autoimmune diseases and inflammatory complications. Since the first successful allo-HSCT was reported in 1968 for the treatment of children with WAS (6), allo-HSCT has gradually developed into a major treatment tool for patients with PIDs (7, 8). The level of immune reconstitution after transplantation is an important factor affecting the prognosis, and nonspecific immune reconstitution is mostly rapid within weeks, while specific immune reconstitution takes 1 year or longer (9, 10), during which time children are highly susceptible to life-threatening serious infections such as bacterial, fungal, and viral infections. It can be seen that robust and timely immune reconstitution is essential for immune protection against opportunistic infections. At present, there are no studies on the differences in immune recovery after transplantation in children with different immunodeficiency diseases, and there is limited published information on immune reconstitution after allo-HSCT in patients with WAS and CGD. Therefore, our study summarized and analyzed the immune reconstitution characteristics of 70 children with WAS and 48 children with CGD within 1 year after undergoing allo-HSCT and summarized transplant-related information in both groups to explore the differences in immune reconstitution in children with both diseases.

## 2 Methods

### 2.1 Cases

A total of 118 children with two PIDs who underwent allo-HSCT at the Transplantation Center of the Department of Hematology-Oncology, Children’s Hospital of Chongqing Medical University from January 2007 to December 2020 were included in the study. Inclusion criteria: (1) having a documented immune reconstitution process on Day 100 posttransplantation or after Day 100 posttransplantation; (2) receiving nondepleted T cells as a conditioning regimen; and (3) undergoing hematopoietic reconstitution. Exclusion criteria: (1) children with failed transplants, failure to achieve hematopoietic reconstitution, or death within 3 months posttransplantation and (2) no record of an immune reconstitution process on or after Day 100 posttransplantation. In the WAS group, the median time to engraftment of neutrophils was Day 11 (5–36 days) posttransplantation, and the median time to engraftment of platelets was Day 20 (5–83 days) posttransplantation. In the CGD group, the median time to engraftment of neutrophils was Day 11 (9–17 days) posttransplantation, and the median time to engraftment of platelets was Day 11 (7–35 days) posttransplantation.

### 2.2 Lymphocyte classification and immunoglobulin monitoring

Changes in lymphocyte subsets, including CD3+ T cells, CD4+ T cells, CD8+ T cells, NK cells (CD56+) and B cells (CD19+), were monitored on Days 15, 30, 100, 180 and 360 after allo-HSCT using flow cytometry. Absolute counts of lymphocyte subpopulations in our center are referenced to previous study (11).

Immunofluorescence detection of immunoglobulin and peptide levels: The levels of various types of immunoglobulins and peptides, including IgM, IgA, IgG, C3, and C4, were detected on Days 15, 30, 100, 180, and 360 after allo-HSCT using an immunoturbidimetric assay.

### 2.3 Statistical processing

SPSS 26.0 was used for statistical processing. The Mann-Whitney U test was used for continuous variables, the chi-square test or Fisher exact test was used for categorical variables, and Student’s t test was used for numerical variables in the comparison of the clinical characteristics of the children. When analyzing the absolute counts of lymphocyte subpopulations in children with both diseases at different time points, data that did not satisfy the normal distribution were analyzed using the Mann-Whitney nonparametric test, and statistical descriptions were performed using the median and interquartile range (Q25 to Q75). Plots were processed using GraphPad Prism 9. P<0.05 (two-sided) was considered a statistically significant difference.

## 3 Results

### 3.1 Basic information and transplant characteristics of children

The basic characteristics of the 118 children are shown in **Table 1**. Among them, children with WAS and CGD were statistically different in terms of the age at transplant, nucleated cell infusion volume, CD34+ cell infusion volume, graft type, and ATG use. The transplantation age of children in the WAS group was apparently younger compared with that in the CGD group; the nucleated cell infusion volume and CD34+ cell infusion volume in the CGD group had been greater compared with that in the WAS group; the proportion of UCBT in the WAS group was apparently higher compared with that in the CGD group; and the proportion of patients receiving ATG pretreatment in the CGD group used to be considerably greater compared with that in the WAS group. There were also differences in infection between the WAS and CGD groups before transplantation: the number of CMV infections in the WAS group was higher than that in the CGD group, and the numbers of EBV and fungal infections in the CGD group were higher than those in the WAS group.The median time to last immunoglobulin infusion after transplantation was 238.50 days (179.75days-329.75days) for children in the WAS group compared to 309.00 days (255.25days-417.75days) for children in the CGD group. The time for last immunoglobulin infusion was significantly less in the WAS group than that in the CGD group after transplantation. All children in the WAS and CGD groups were male, 1 child in the WAS group was transplanted with CB+BM, and the rest were transplanted with a single graft. As of December 31, 2022, one child in the WAS group died of respiratory and circulatory failure due to severe pulmonary infection in the 11th month after allo-HSCT (2013/7/2), and the remaining 117 children survived. The median follow-up time was 78.1 months (24.4-159.3 months) for the surviving children in the WAS group (69 children) and 52.3 months (19.5-128.0 months) for the surviving children in the CGD group (48 children).

**Table 1:** Basic characteristics and transplantion characteristics of 118 children in the WAS and CGD groups.

Clinical Information	Wiskott-Aldrich syndrome (70 cases)	Chronic granulomatous disease (48 cases)	P-value
Patient sex[Number of cases(%)]			
Male	70 (100)	48 (100)	/
**Median age at transplantation[M(range)]**	16.88months (7.00months-105.40months)	28.48months (8.53months-117.43months)	0.002**
**Nucleated cell infusion volume** **[×10^8^/kg, M (range)]**	9.420 (0.420-24.870)	10.600 (1.344-28.000)	0.026*
**CD34+ cell infusion volume** **[×10^6^/kg, M (range)]**	5.44 (0.036-25.375)	6.710 (0.479-19.270)	0.017*
**HLA compatibility [cases (%)]**			**0.254**
HLA match (10/10 match or 6/6 match)	29 (41)	25 (52)	
HLA mismatch	41 (58)	23 (48)	
**ABO matching [cases (%)]**			**0.379**
Matched	25 (36)	21 (44)	
Mismatch	45 (64)	27 (56)	
**Graft type [cases (%)]**			<0.001**
CB	24 (34)	1 (2)	
Non-CB(PB or BM or CB+BM)	46 (64)	47 (98)	
**Donor type [cases (%)]**			**0.686**
URD	53 (76)	31 (65)	
sibling donor	12 (17)	12 (25)	
Paternal or maternal donor	5 (7)	5 (10)	
**Conditioning regimen [cases (%)]**			0.002**
Using ATG	32 (46)	36 (75)	
Not using ATG	38 (54)	12 (25)	
**GVHD prophylaxis [cases (%)]**			0.612
CSA	1 (2)	0 (0)	
CSA+MP	24 (34)	3 (6)	
CSA+MTX	17 (24)	4 (8)	
CSA+MMF	26 (37)	35 (73)	
CSA+MTX+MMF	2 (3)	6 (13)	
**aGVHD [cases (%)]**			0.480
None	15 (21)	17 (36)	
Grade I	15 (21)	15 (31)	
Grade II	35 (50)	15 (31)	
Grade III	5 (8)	1 (2)	
**cGVHD [cases (%)]**			0.494
cGVHD occurred	22 (31)	18 (38)	
No cGVHD occurred	48 (69)	30 (62)	
Infection before allo-HSCT [cases (%)]			
CMV infection (+)	22 (31)	7 (15)	0.037*
EBV infection (+)	14 (20)	19 (40)	0.020*
Fungus	12 (17)	33 (69)	<0.001**
**Time of last gammaglobulin infusion after transplantation [M(range)]**	238.50days (179.75days-329.75days)	309.00days (255.25days-417.75days)	0.002**

HLA indicates human leukocyfe antigen; CB indicates cord blood; PB indicates peripheral blood;BM indicates bone marrow; URD indicates unrelated donor; ATG indicates antithymocyte globulin; GVHD indicates graft versus host disease; CSA indicates cyclosporine; MP indicates methylprednisolone; MTX indicates methotrexate; MMF indicates mycophenolate mofetil; CMV indicates cytomegalovirus. “HLA compatibility” refers to either HLA 10/10 loci match or HLA 6/6 loci match; with one child in the WAS group being HLA 6/6 loci match and the remaining children with full compatibility being HLA 10/10 loci match. “CMV/EBV infection” is defined as “DNAemia”. It refers to the detection of CMV/EBV DNA in whole blood in amounts greater than or equal to 400 copies/ml. *P<0.05, **P<0.01, the difference is statistically significant.

### 3.2 Reconstruction of lymphocyte subsets in the WAS and CGD groups

**Figure 1:**
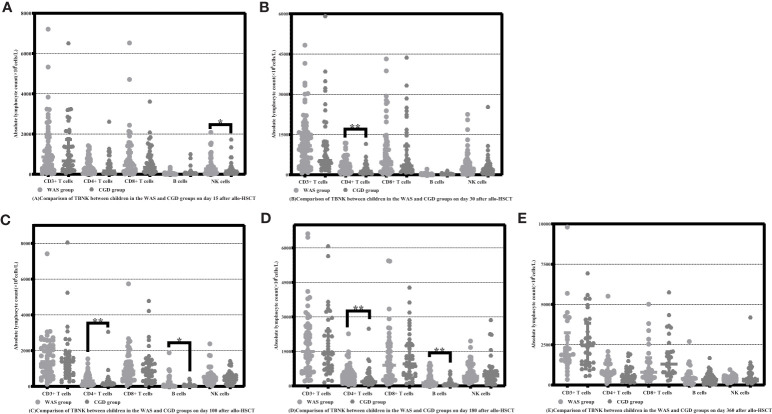
Comparison of changes in the number of lymphocyte subpopulations in children in the WAS and CGD groups at different time points after allo-HSCT. The time points included day 15, day 30, day 100, day 180, and day 360 after transplantation in the WAS and CGD groups, and the lymphocyte categories included CD3+ T cells, CD4+ T cells, CD8+ T cells, B cells, and NK cells. **(A–E)** Comparison of lymphocyte subsets count in WAS group and CGD group on Days 15, 30, 100, 180 and 360 after allo-HSCT. *P<0.05, **P<0.01. Horizontal bars represent median with interquartile range **(A–E)**.

On Day 15 after allo-HSCT, the WAS group had significantly higher NK cell counts than the CGD group [233.47 (59.69-395.78) ×10^6^cells/L vs. 129.25 (28.41-217.53) ×10^6^cells/L, P=0.022]. On Days 30, 100 and 180 after allo-HSCT, the WAS group had notably higher CD4+ T-cell counts than the CGD group [305.47 (117.81-427.50) ×10^6^cells/L vs. 134.77 (62.74-244.97) ×10^6^cells/L, P<0.001], [312.80 (127.00-546.95) ×10^6^cells/L vs. 148.64 (111.06-282.32) ×10^6^cells/L, P=0.005], [397.93 (228.15-631.74) ×10^6^cells/L vs. 179.85(120.61-366.11)×10^6^cells/L, P=0.004]. On Days 100 and 180 after allo-HSCT, the WAS group had considerably higher B-cell counts than the CGD group [32.28 (12.24-231.74) ×10^6^cells/L vs. 18.43 (8.39-55.82) ×10^6^cells/L, P=0.027], [132.05 (43.04-418.20) ×10^6^cells/L vs. 37.32 (14.96-132.36) ×10^6^cells/L, P=0.002] shown in **Table 2** and **Figures 1A–E**.

**Table 2:** Changes in lymphocyte subpopulation counts after allo-HSCT in WAS and CGD groups [×10^6^cells/L, P50 (P25, P75)].

Lymphocyte subsets	WAS group (with numerical examples)	CGD group (with numerical examples)	Z-value	P-value
**Day 15 after transplantation**	(49 cases)	(46 cases)		
CD3+T cells	859.82(283.60-1992.65)	661.52(233.43-1729.73)	-0.380	0.704
CD4+T cells	258.00(92.44-695.20)	166.79(55.33-519.42)	-0.894	0.372
CD8+T cells	445.18(156.52-1085.45)	344.22(123.61-952.20)	-0.328	0.743
B cells	8.38(2.53-39.46)	13.43(4.37-38.77)	-1.005	0.315
NK cells	233.47(59.69-395.78)	129.25(28.41-217.53)	-2.294	0.022*
**Day 30 after transplantation**	(63 cases)	(44 cases)		
CD3+T cells	1089.14(397.80-1704.00)	534.28(297.38-1229.77)	-1.608	0.108
CD4+T cells	305.47(117.81-427.50)	134.77(62.74-244.97)	-3.596	<0.001**
CD8+T cells	477.31(159.89-1107.60)	330.63(146.63-1072.77)	-1.070	0.285
B cells	9.59(2.92-21.96)	4.74(2.27-16.28)	-1.656	0.098
NK cells	208.87(109.56-472.97)	163.62(64.91-337.45)	-1.538	0.124
**Day 100 after transplantation**	(55 cases)	(37 cases)		
CD3+T cells	1385.10(809.40-2262.91)	1360.22(647.97-1865.37)	-0.816	0.414
CD4+T cells	312.80(127.00-546.95)	148.64(111.06-282.32)	-2.839	0.005**
CD8+T cells	854.41(404.59-1372.92)	833.04(357.11-1383.93)	-0.123	0.902
B cells	32.28(12.24-231.74)	18.43(8.39-55.82)	-2.206	0.027*
NK cells	421.80(261.30-595.74)	424.51(228.43-587.48)	-0.012	0.990
**Day 180 after transplantation**	(56 cases)	(40 cases)		
CD3+T cells	1495.38(635.25-2543.50)	1402.72(772.59-2381.62)	-0.089	0.929
CD4+T cells	397.93(228.15-631.74)	179.85(120.61-366.11)	-2.913	0.004**
CD8+T cells	895.22(267.68-1713.58)	959.78(466.00-1843.30)	-0.922	0.357
B cells	132.05(43.04-418.20)	37.32(14.96-132.36)	-3.144	0.002**
NK cells	383.40(247.00-848.98)	463.20(198.24-618.66)	-0.565	0.572
**Day 360 after transplantation**	(26 cases)	(35 cases)		
CD3+T cells	1882.78(1444.52-3239.87)	2437.47(1266.62-3833.13)	-0.263	0.793
CD4+T cells	761.42(530.07-1338.72)	521.35(283.83-994.47)	-1.925	0.054
CD8+T cells	788.68(473.49-1690.43)	1291.43(707.91-2060.67)	-1.809	0.070
B cells	393.18(177.30-701.23)	334.85(135.91-608.59)	-0.963	0.336
NK cells	373.87(235.00-548.36)	306.73(247.19-729.00)	-0.190	0.850

*P<0.05, **P<0.01, the difference is statistically significant.

### 3.3 The reconstitution of lymphocyte subsets and analysis of graft types in children aged 1-3 years in the WAS and CGD groups

We selected the age group with the largest number of transplants in WAS and CGD groups, i.e., children aged 1-3 years who underwent allo-HSCT, and we analyzed the reconstruction of lymphocyte subpopulations in these children at different time points after allo-HSCT. The results are as follows.

On Day 15 posttransplantation, NK cell counts in the WAS group were considerably higher than those in the CGD group among children aged 1-3 years who underwent transplants [265.60 (61.98-375.97) ×10^6^cells/L vs. 113.46 (26.85-223.29) ×10^6^cells/L, P=0.050]. On Days 30 and 180 posttransplantation, the WAS group had notably higher CD4+ T-cell counts than the CGD group among children aged 1-3 years who underwent transplants [325.80 (143.29-465.00) ×10^6^cells/L vs. 133.93 (63.94-241.75) ×10^6^cells/L, P=0.011], [308.20 (201.21-526.40) ×10^6^cells/L vs. 174.39 (129.96-282.96) ×10^6^cells/L, P=0.025]. On Day 180 posttransplantation, B-cell counts in the WAS group were consistently higher than those in the CGD group among children aged 1-3 years who underwent transplants [131.20 (95.70-266.64) ×10^6^cells/L vs. 28.02 (13.10-104.23) ×10^6^cells/L, P=0.003]. On Day 360 posttransplantation, the CGD group had notably higher CD8+ T-cell counts than the WAS group among children aged 1-3 years who underwent transplants [1278.28 (674.62-3540.85)×10^6^cells/L vs. 578.54 (454.63-803.18)×10^6^cells/L, P=0.021].

**Figure 2:**
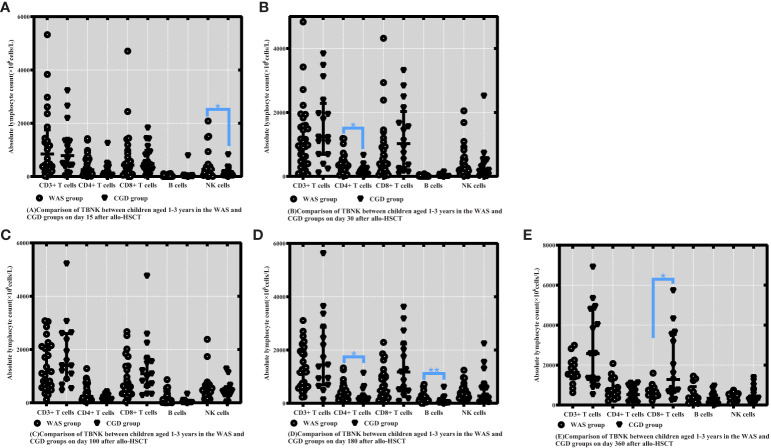
Comparison of changes in lymphocyte subpopulation values at different time points after allo-HSCT in children aged 1-3 years in the WAS and CGD groups. The time points included day 15, day 30, day 100, day 180, and day 360 after transplantation, and the lymphocyte categories included CD3+ T cells, CD4+ T cells, CD8+ T cells, B cells, and NK cells. **(A–E)** Comparison of lymphocyte subsets count between children aged 1-3 years in the WAS and CGD groups on Days 15, 30, 100, 180 and 360 after allo-HSCT. *P<0.05, **P<0.01. Horizontal bars represent median with interquartile range **(A-E)**.

In addition, the study compared the graft types of children in the WAS and CGD groups who underwent allo-HSCT at the age of 1-3 years. The results showed that there were no significant differences in graft type between children in the WAS and CGD groups who underwent allo-HSCT at the age of 1-3 years (P=0.100) shown in **Tables 3**, **4**, **Figures 2A–E**.

**Table 3:** Changes in lymphocyte subsets after allo-HSCT in children aged 1-3 years in the WAS and CGD groups[×10^6^cells/L, P50 (P25, P75)].

Lymphocyte subsets	WAS group (with numerical examples)	CGD group (with numerical examples)	Z-value	P-value
**Day 15 after transplantation**	(24 cases)	(18 cases)		
CD3+T cells	848.02 (315.17-1754.27)	784.76 (412.92-1348.47)	-0.178	0.859
CD4+T cells	255.40 (103.02-711.60)	115.66 (49.10-354.99)	-1.500	0.134
CD8+T cells	427.59 (142.14-951.58)	486.76 (254.07-984.84)	-0.330	0.741
B cells	17.16 (2.02-49.28)	13.14 (4.59-33.86)	-0.381	0.703
NK cells	265.60 (61.98-375.97)	113.46 (26.85-223.29)	-1.957	0.050*
**Day 30 after transplantation**	(33 cases)	(16 cases)		
CD3+T cells	955.50 (434.79-1589.08)	1226.86 (685.96-2283.40)	-1.279	0.201
CD4+T cells	325.80 (143.29-465.00)	133.93 (63.94-241.75)	-2.558	0.011*
CD8+T cells	420.00 (176.11-939.12)	1023.30 (277.45-2029.93)	-1.748	0.080
B cells	9.46 (2.85-24.51)	5.60 (3.31-22.23)	-0.405	0.685
NK cells	208.87 (127.66-498.09)	194.77 (66.88-504.44)	-0.618	0.536
**Day 100 after transplantation**	(26 cases)	(15 cases)		
CD3+T cells	1138.14 (569.03-2123.91)	1400.23 (877.25-2603.69)	-1.056	0.291
CD4+T cells	237.67 (116.21-593.86)	179.05 (114.65-274.89)	-1.164	0.244
CD8+T cells	616.96 (351.32-1375.07)	1088.66 (613.11-1674.79)	-1.380	0.167
B cells	30.19 (11.33-289.29)	14.48 (6.03-28.53)	-1.813	0.070
NK cells	479.32 (295.80-630.54)	455.72 (306.86-530.48)	-0.514	0.607
**Day 180 after transplantation**	(27 cases)	(15 cases)		
CD3+T cells	1172.03 (592.80-1955.74)	1440.82 (687.55-2860.71)	-1.299	0.194
CD4+T cells	308.20 (201.21-526.40)	174.39 (129.96-282.96)	-2.244	0.025*
CD8+T cells	474.83 (243.60-1016.59)	1177.41 (462.49-2231.12)	-1.798	0.072
B cells	131.20 (95.70-266.64)	28.02 (13.10-104.23)	-2.979	0.003**
NK cells	351.27 (232.30-536.00)	273.23 (58.28-731.15)	-0.276	0.783
**Day 360 after transplantation**	(14 cases)	(15 cases)		
CD3+T cells	1630.57 (1318.76-2162.73)	2570.91 (1157.66-4802.66)	-0.829	0.407
CD4+T cells	734.61 (458.72-1322.85)	521.35 (239.17-994.47)	-1.353	0.176
CD8+T cells	578.54 (454.63-803.18)	1278.28 (674.62-3540.85)	-2.313	0.021*
B cells	369.88 (177.30-923.94)	342.08 (85.87-608.59)	-1.266	0.206
NK cells	483.50 (229.56-578.26)	306.73 (248.12-794.20)	-0.218	0.827

*P<0.05, **P<0.01, *P=0.050 indicates marginal significant.

**Table 4:** Types of grafts in children in the WAS and CGD groups who received allo-HSCT at the age of 1-3 years.

	Wiskott-Aldrich syndrome (34 cases)	Chronic granulomatous disease (18 cases)	P-value
**Graft type [cases (%)]**			0.100
CB	10 (29)	1 (6)	
Non-CB(PB or BM)	24 (71)	17 (94)	

CB indicates cord blood; PB indicates peripheral blood; BM indicates bone marrow.

### 3.4 Reconstruction of lymphocyte subsets after umbilical cord blood transplantation and non-UCBT in the WAS group

**Figure 3:**
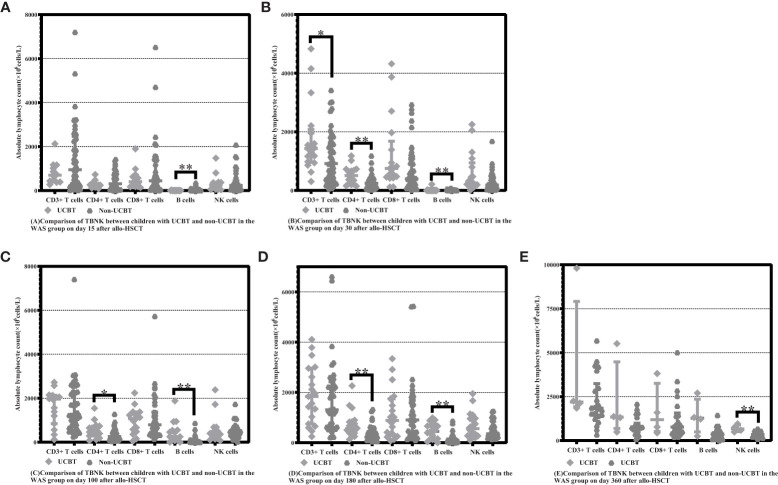
Comparison of changes in lymphocyte subpopulation values at different time points after allo-HSCT in children with UCBT and non-UCBT in the WAS group. The time points included day 15, day 30, day 100, day 180, and day 360 after transplantation, and the lymphocyte categories included CD3+ T cells, CD4+ T cells, CD8+ T cells, B cells, and NK cells. **(A–E)** Comparison of lymphocyte subsets count between children with UCBT and non- UCBT in WAS group on Days 15, 30, 100, 180 and 360 after allo-HSCT. *P<0.05, **P<0.01. Horizontal bars represent median with interquartile range **(A–E)**.

On Days 15 and 30 posttransplantation, children who underwent non-UCBT had significantly higher B-cell counts than children who underwent UCBT in the WAS group [15.55 (5.69-60.66) ×10^6^cells/L vs. 0.48 (0.00-1.41) ×10^6^cells/L, P<0.001], [14.63 (8.48-25.46) ×10^6^cells/L vs. 1.24 (0.20-2.23) ×10^6^cells/L, P<0.001]. On Days 100 and 180 posttransplantation, children who underwent UCBT had apparently higher B-cell counts than children who underwent non-UCBT in the WAS group [250.80 (125.50-552.30) ×10^6^cells/L vs. 24.99 (11.18-89.16) ×10^6^cells/L, P=0.001], [445.600 (131.20-712.71) ×10^6^cells/L vs. 96.60 (39.24-191.95) ×10^6^cells/L, P<0.001]. On Day 30 posttransplantation, children who underwent UCBT had notably higher CD3+ T-cell counts than children who underwent non-UCBT in the WAS group [1427.40 (961.69-2080.18) ×10^6^cells/L vs. 914.92 (282.17-1614.18) ×10^6^cells/L, P=0.026]. On Days 30, 100 and 180 posttransplantation, children who underwent UCBT had obviously higher CD4+ T-cell counts than children who underwent non-UCBT in the WAS group [507.04 (218.31-705.45) ×10^6^cells/L vs. 244.39 (97.21-401.69) ×10^6^cells/L, P=0.003], [466.82 (289.30-734.57) ×10^6^cells/L vs. 260.25 (118.72-479.53) ×10^6^cells/L, P=0.038], [571.29 (384.52-832.00) ×10^6^cells/L vs. 308.20 (168.48-488.70) ×10^6^cells/L, P=0.002]. On Day 360 posttransplantation, children who underwent UCBT had markedly higher NK cell counts than children who underwent non-UCBT in the WAS group [668.62 (515.46-901.64) ×10^6^cells/L vs. 346.13 (229.56-488.45) ×10^6^cells/L, P=0.007] shown in **Table 5** and **Figures 3A–E**.

**Table 5:** Changes in lymphocyte subpopulation counts after UCBT and non-UCBT in children in the WAS group[×10^6^cells/L, P50 (P25, P75)].

Lymphocyte subsets	UCBT (with numerical examples)	Non-UCBT(with numerical examples)	Z-value	P-value
**Day 15 after transplantation**	(12 cases)	(37 cases)		
CD3+T cells	702.07 (400.10-1126.10)	950.81 (213.43-2464.90)	-0.558	0.577
CD4+T cells	252.90 (161.82-307.74)	310.47 (55.15-775.54)	-0.465	0.642
CD8+T cells	403.92 (181.96-699.86)	445.18 (134.99-1407.00)	-0.023	0.981
B cells	0.48 (0.00-1.41)	15.55 (5.69-60.66)	-4.698	<0.001**
NK cells	249.35 (57.77-375.97)	233.47 (63.09-438.78)	-0.023	0.981
**Day 30 after transplantation**	(17 cases)	(46 cases)		
CD3+T cells	1427.40 (961.69-2080.18)	914.92 (282.17-1614.18)	-2.230	0.026*
CD4+T cells	507.04 (218.31-705.45)	244.39 (97.21-401.69)	-2.989	0.003**
CD8+T cells	748.80 (442.73-1680.34)	391.96 (145.24-1090.25)	-1.928	0.054
B cells	1.24 (0.20-2.23)	14.63 (8.48-25.46)	-4.770	<0.001**
NK cells	234.00(191.07-943.95)	186.21 (83.18-447.53)	-1.827	0.068
**Day 100 after transplantation**	(15 cases)	(40 cases)		
CD3+T cells	1964.00 (841.30-2133.50)	1246.57 (693.53-2300.73)	-0.170	0.865
CD4+T cells	466.82 (289.30-734.57)	260.25 (118.72-479.53)	-2.079	0.038*
CD8+T cells	1083.15 (483.30-1374.80)	789.17 (365.04-1367.76)	-0.283	0.777
B cells	250.80 (125.50-552.30)	24.99 (11.18-89.16)	-3.326	0.001**
NK cells	358.00 (167.04-686.40)	440.02 (267.63-583.94)	-0.605	0.545
**Day 180 after transplantation**	(19 cases)	(37 cases)		
CD3+T cells	1838.58 (746.30-2961.00)	1305.60 (616.29-2417.93)	-0.857	0.392
CD4+T cells	571.29 (384.52-832.00)	308.20 (168.48-488.70)	-3.072	0.002**
CD8+T cells	885.50 (296.40-1744.41)	904.93 (256.35-1686.01)	-0.043	0.965
B cells	445.600 (131.20-712.71)	96.60 (39.24-191.95)	-3.591	<0.001**
NK cells	556.60 (267.30-966.31)	354.20 (243.35-621.13)	-1.272	0.203
**Day 360 after transplantation**	(4 cases)	(22 cases)		
CD3+T cells	2191.66 (1922.02-7913.82)	1843.55 (1363.38-3239.88)	-1.208	0.227
CD4+T cells	1336.91 (694.57-4475.28)	746.18 (509.41-993.53)	-1.279	0.201
CD8+T cells	1187.23 (523.46-3258.09)	771.69 (473.49-1570.69)	-0.640	0.522
B cells	1272.45 (495.28-2353.98)	374.38 (160.30-554.29)	-1.919	0.055
NK cells	668.62 (515.46-901.64)	346.13 (229.56-488.45)	-2.701	0.007**

*P<0.05, **P<0.01, the difference is statistically significant.

### 3.5 Reconstruction of lymphocyte subsets after non-UCBT in the WAS and CGD groups

**Figure 4:**
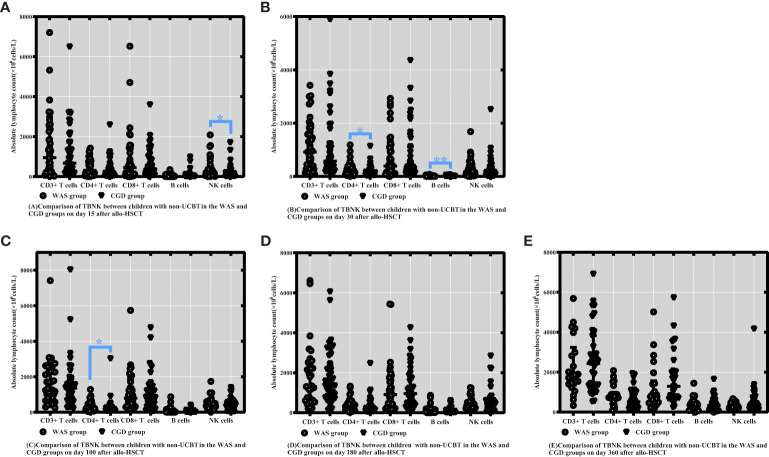
Comparison of changes in lymphocyte subpopulation values at different time points after allo-HSCT in children with non-UCBT in the WAS and CGD groups. The time points included day 15, day 30, day 100, day 180, and day 360 after transplantation, and the lymphocyte categories included CD3+ T cells, CD4+ T cells, CD8+ T cells, B cells, and NK cells. **(A–E)** Comparison of lymphocyte subsets count between children with non-UCBT in WAS and CGD groups on Days 15, 30, 100, 180 and 360 after allo-HSCT. *P<0.05, **P<0.01. Horizontal bars represent median with interquartile range **(A-E)**.

On Day 15 posttransplantation, NK cell counts were probably higher in the non-cord-blood-transplanted children with WAS compared to the non-cord-blood-transplanted children with CGD [233.47 (63.09-438.78) ×10^6^cells/L vs. 129.69 (28.02-222.26) ×10^6^cells/L, P=0.043]. On Days 30 and 100 posttransplantation, CD4+ T-cell counts were significantly higher in the non-cord-blood-transplanted children with WAS compared to the non-cord-blood-transplanted children with CGD [244.39 (97.21-401.69) ×10^6^cells/L vs. 139.22 (63.26-253.12) ×10^6^cells/L, P=0.024], [260.25 (118.72-479.53) ×10^6^cells/L vs. 148.64 (111.06-282.32) ×10^6^cells/L, P=0.049]. On Day 30 posttransplantation, B-cell counts were notably higher in the non-cord-blood-transplanted children with WAS compared to the non-cord-blood-transplanted children with CGD [14.63 (8.48-25.46) ×10^6^cells/L vs. 4.51 (2.21-11.52) ×10^6^cells/L, P<0.001] shown in **Table 6** and **Figures 4A–E**.

**Table 6:** Changes of lymphocyte subsets after non-UCBT in WAS and CGD groups[×10^6^cells/L, P50 (P25, P75)].

Lymphocyte subsets	WAS group non-UCBT (with numerical examples)	CGD group non-UCBT (with numerical examples)	Z-value	P-value
**Day 15 after transplantation**	(37 cases)	(45 cases)		
CD3+T cells	950.81 (213.43-2464.90)	667.98 (272.82-1729.74)	-0.368	0.713
CD4+T cells	310.47 (55.15-775.55)	173.11 (67.09-538.19)	-0.666	0.505
CD8+T cells	445.178 (134.99-1407.00)	345.25 (137.57-970.20)	-0.154	0.878
B cells	15.55 (5.69-60.66)	13.69 (4.63-39.46)	-0.731	0.464
NK cells	233.47 (63.09-438.78)	129.69 (28.02-222.26)	-2.027	0.043*
**Day 30 after transplantation**	(46 cases)	(43 cases)		
CD3+T cells	914.92 (282.17-1614.18)	576.61 (312.84-1232.69)	-0.435	0.663
CD4+T cells	244.39 (97.21-401.69)	139.22 (63.26-253.12)	-2.25	0.024*
CD8+T cells	391.97 (145.24-1090.25)	341.00 (149.49-1122.24)	-0.115	0.908
B cells	14.63 (8.48-25.46)	4.51 (2.21-11.52)	-4.097	<0.001**
NK cells	186.21 (83.18-447.53)	167.40 (70.71-342.32)	-0.608	0.543
**Day 100 after transplantation**	(40 cases)	(37 cases)		
CD3+T cells	1246.57 (693.53-2300.73)	1360.22 (647.97-1865.37)	-0.54	0.589
CD4+T cells	260.25 (118.72-479.53)	148.64 (111.06-282.32)	-1.968	0.049*
CD8+T cells	789.17 (365.04-1367.76)	833.04 (357.11-1383.93)	-0.051	0.959
B cells	24.99 (11.18-89.16)	18.43 (8.39-55.82)	-0.943	0.346
NK cells	440.02 (267.63-583.94)	424.51 (228.43-587.48)	-0.112	0.911
**Day 180 after transplantation**	(37 cases)	(40 cases)		
CD3+T cells	1305.60 (616.29-2417.93)	1402.72 (772.59-2381.62)	-0.285	0.775
CD4+T cells	308.20 (168.48-488.70)	179.85 (120.61-366.11)	-1.611	0.107
CD8+T cells	904.93 (256.35-1686.01)	959.78 (466.00-1843.30)	-0.877	0.381
B cells	96.60 (39.24-191.95)	37.32 (14.96-132.36)	-1.621	0.105
NK cells	354.20 (243.35-621.13)	463.20 (198.24-618.66)	-0.02	0.984
**Day 360 after transplantation**	(22 cases)	(35 cases)		
CD3+T cells	1843.55 (1363.38-3239.87)	2298.37 (1266.62-3833.13)	-0.475	0.635
CD4+T cells	746.18 (509.41-993.54)	480.49 (283.83-963.09)	-1.442	0.149
CD8+T cells	771.69 (473.49-1570.69)	1291.43 (707.91-2060.67)	-1.895	0.058
B cells	374.38 (160.30-554.29)	275.90 (122.40-561.28)	-0.295	0.768
NK cells	346.13 (229.56-488.45)	301.88 (194.15-729.00)	-0.77	0.441

*P<0.05, **P<0.01, the difference is statistically significant.

### 3.6 Immunoglobulin reconstitution in the WAS and CGD groups

**Figure 5:**
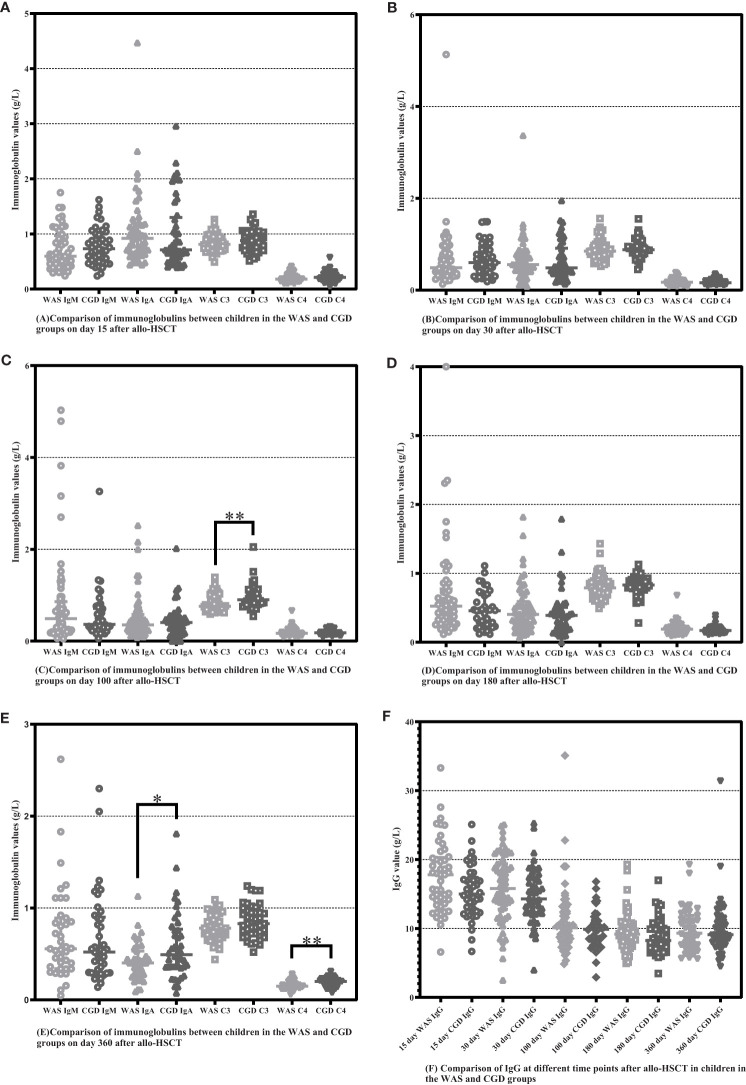
Comparison of immunoglobulins between WAS and CGD groups at different time points after allo-HSCT. The time points included day 15, day 30, day 100, day 180, and day 360 after transplantation, and various immunoglobulins include IgM, IgA, IgG, C3 and C4. **(A–F)** Comparison of immunoglobulins between WAS and CGD groups on Days 15, 30, 100, 180 and 360 after allo-HSCT. *P < 0.05, **P < 0.01. Horizontal bars represent median with interquartile range **(A-F)**.

On Day 100 after allo-HSCT, the CGD group had higher C3 levels than the WAS group [0.9000 (0.8075-1.0750) ×g/L vs. 0.7650 (0.6900-0.9825) ×g/L, P=0.002]. On Day 360 after allo-HSCT, the CGD group had higher IgA and C4 levels than the WAS group [0.4930 (0.3595-0.76975) ×g/L vs. 0.4030 (0.2740-0.5320) ×g/L, P=0.033], [0.2000 (0.1475-0.2200) ×g/L vs. 0.150 (0.130-0.180) ×g/L, P=0.004]. There was no statistically significant change in immunoglobulin levels between the both groups on Days 15, 30 and 180 after allo-HSCT (P > 0.05). Among the patients who underwent allo-HSCT in our center, due to the influence of regular infusions of gamma globulin (IVIG), the IgG levels of the WAS and CGD groups of patients at various time points posttransplantation are not of comparative value shown in **Table 7** and **Figures 5A–F**.

**Table 7:** Changes in immunoglobulin levels after allo-HSCT in the WAS and CGD groups [g/L, P50 (P25, P75)].

Immunoglobulin	WAS group (with numerical examples)	CGD group (with numerical examples)	Z-value	P-value
**Day 15 after transplantation**	**(49 cases)**	**(45 cases)**		
IgM	0.5950(0.4365-0.9690)	0.7310(0.4955-0.9985)	-1.026	0.305
IgA	0.9180(0.6505-1.1900)	0.7110(0.5750-1.3000)	-1.211	0.226
IgG	17.8000(13.5000-20.3000)	15.0000(12.6500-17.1500)	-1.934	0.053
C3	0.8100(0.7200-0.9400)	0.8800(0.7100-1.0300)	-0.746	0.456
C4	0.1800(0.1500-0.2100)	0.2100(0.1600-0.2750)	-1.289	0.197
**Day 30 after transplantation**	(59 cases)	(46 cases)		
IgM	0.4880(0.3520-0.8100)	0.6010(0.3655-0.9375)	-0.891	0.373
IgA	0.5560(0.4070-0.7600)	0.4865(0.3575-0.91325)	-0.394	0.694
IgG	15.800(12.800-19.400)	14.300(12.2750-17.4250)	-1.392	0.164
C3	0.850(0.7300-1.000)	0.8800(0.7875-0.9900)	-1.037	0.300
C4	0.1700(0.1400-0.2100)	0.1600(0.1300-0.2125)	-0.071	0.943
**Day 100 after transplantation**	(66 cases)	(42 cases)		
IgM	0.49800(0.22125-0.85375)	0.36250(0.26375-0.71275)	-0.573	0.566
IgA	0.34(0.22-0.56)	0.39(0.16-0.51)	-0.252	0.801
IgG	10.1500(8.3425-12.4250)	9.8850(7.9150-11.1500)	-1.027	0.304
C3	0.7650(0.6900-0.9825)	0.9000(0.8075-1.0750)	-3.105	0.002**
C4	0.1700(0.1500-0.2400)	0.1800(0.1475-0.2325)	-0.148	0.882
**Day 180 after transplantation**	(58 cases)	(41 cases)		
IgM	0.51600(0.28225-0.76175)	0.4570(0.2265-0.6895)	-1.755	0.079
IgA	0.40000(0.24175-0.57550)	0.3900(0.1680-0.5060)	-0.970	0.332
IgG	9.1700(8.0200-10.7000)	8.2500(6.9700-10.2500)	-1.321	0.186
C3	0.7900(0.6850-0.8975)	0.830(0.750-0.915)	-0.998	0.318
C4	0.1900(0.1400-0.2225)	0.170(0.140-0.200)	-1.075	0.282
**Day 360 after transplantation**	(39 cases)	(42 cases)		
IgM	0.5560(0.3390-0.8920)	0.52150(0.29475-0.89975)	-0.581	0.561
IgA	0.4030(0.2740-0.5320)	0.4930(0.3595-0.76975)	-2.136	0.033*
IgG	9.32(7.36-11.90)	9.100(7.955-11.575)	-0.076	0.940
C3	0.78(0.67-0.92)	0.8300(0.7200-0.9775)	-1.929	0.054
C4	0.150(0.130-0.180)	0.2000(0.1475-0.2200)	-2.896	0.004**

*P value <0.05, **P value <0.01, the difference is statistically significant.

### 3.7 Infection and GVHD after allo-HSCT in the WAS and CGD groups

In this paper, children in the WAS and CGD groups were divided into different age groups (<1 year, 1-3 years, 3-5 years, and >5 years), and we compared the numbers of posttransplantation infections and occurrences of aGVHD in children in different age groups (shown in **Table 8**). In children younger than 1 year of age, the numbers of posttransplantation EBV and fungal infections were higher in the CGD group than in the WAS group. In children 1-3 years of age, the numbers of posttransplantation EBV and CMV infections were higher in the CGD group than in the WAS group. In children older than 5 years, the number of posttransplantation fungal infections was higher in the CGD group than in the WAS group. When comparing the incidence of aGVHD in children of different ages in the two groups, the results showed no statistical difference in the incidence of aGVHD in children of all ages in the WAS and CGD groups (P > 0.05). In **Table 1**, we also found that the incidence of cGVHD after transplantation in children with WAS and CGD was also not statistically different.

**Table 8:** Incidence of post-transplant infection and aGVHD in children of different ages in the WAS and CGD groups.

	WAS[%(Number of positives/total)]	CGD[%(Number of positives/total)]	P-value
Younger than 1 year old			
CMV infection after transplantation(+)	59 (13/22)	55 (6/11)	1.000
EBV infection after transplantation(+)	64 (14/22)	100 (11/11)	0.031*
aGVHD	68 (15/22)	82 (9/11)	0.681
septicemia	82 (18/22)	82 (9/11)	1.000
fungus	41 (9/22)	91 (10/11)	0.009**
other	32 (7/22)	27 (3/11)	1.000
1-3 year old			
CMV infection after transplantation(+)	41 (14/34)	72 (13/18)	0.033*
EBV infection after transplantation(+)	62 (21/34)	89 (16/18)	0.040*
aGVHD	82 (28/34)	61 (11/18)	0.092
septicemia	79 (27/34)	72 (13/18)	0.558
fungus	59 (20/34)	83 (15/18)	0.138
other	18 (6/34)	22 (4/18)	0.977
3-5 year old			
CMV infection after transplantation(+)	29 (2/7)	56 (5/9)	0.358
EBV infection after transplantation(+)	57 (4/7)	78 (7/9)	0.596
aGVHD	86 (6/7)	67 (6/9)	0.585
septicemia	57 (4/7)	89 (8/9)	0.262
fungus	71 (5/7)	100 (9/9)	0.175
other	29 (2/7)	22 (2/9)	1.000
Older than 5 year old			
CMV infection after transplantation(+)	57 (4/7)	60 (6/10)	1.000
EBV infection after transplantation(+)	71 (5/7)	90 (9/10)	0.537
aGVHD	86 (6/7)	50 (5/10)	0.304
septicemia	86 (6/7)	80 (8/10)	1.000
fungus	14 (1/7)	90 (9/10)	0.004**
other	57 (4/7)	20 (2/10)	0.162

Other: includes various bacteria, syncytial viruses, influenza/parainfluenza viruses, BK viruses, adenoviruses, etc. *P<0.05, **P<0.01, the difference is statistically significant.

## 4 Discussion

The effect of allo-HSCT depends primarily on the hematopoietic and immune reconstitution capacity of the donor’s hematopoietic stem cells in the recipient, which suggests that posttransplantation hematopoietic and immune reconstitution is the basis of successful allo-HSCT. It was in this study that we compared for the first time the immune reconstitution data of WAS and CGD within 1 year after allo-HSCT. We first analyzed the reconstitution of lymphocyte subsets after transplantation in children with WAS and CGD by flow cytometry and found that the reconstitution of lymphocyte subsets posttransplantation was different in both groups. At Day 15 after allo-HSCT, the WAS group had significantly higher NK cell counts compared to the CGD group. At Days 30, 100, and 180 posttransplantation, the WAS group had notably higher CD4+ T-cell counts compared to the CGD group. At Days 100 and 180 posttransplantation, the WAS group had considerably higher B-cell counts compared to the CGD group. In general, immune reconstitution in the WAS group was faster than that in the CGD group after allo-HSCT.

WAS group has faster immune reconstitution than CGD group after allo-HSCT, which may be attributed to different factors inherent in the different primary diseases or to some modifiable factors (e.g., graft type). First, we included factors such as age at transplantation, graft type, HLA match, conditioning regimen, graft MNC content, and graft CD34+ cell count as independent variables, and the absolute counts of lymphocyte subpopulations on days 15, 30, 100, 180, and 360 after transplantation were selected as the dependent variables for multiple linear regression analysis, respectively. The results showed that donor type, HLA match, age at transplantation, posttransplantion CMV and EBV infection, graft type, conditioning regimen, aGVHD and blood group could affect the absolute counts of lymphocyte subpopulations.

Then, to investigate the reasons for the faster immune reconstitution in the WAS group than in the CGD group, we compared the basic clinical characteristics of the two groups of children (**Table 1**), including the age at transplantation, graft mononuclear cell (MNC) content and CD34+ cell count, human leukocyte antigen (HLA) matching, ABO blood group, graft type, donor type, conditioning regimen, graft versus host disease (GVHD) prophylaxis regimen, and GVHD occurrence. The graft type, graft MNC content, CD34+ cell count, and conditioning regimen were significantly different between the WAS and CGD groups. It is noteworthy that in this study, there were more umbilical cord blood transplantations (UCBTs) in the WAS group and only one UCBT in the CGD group, which led to differences in graft type between the two groups. More children with WAS underwent UCBT, while cord blood (CB) contained fewer cells, which led to differences in graft MNC content and CD34+ cell counts between children with WAS and children with CGD. The lower number of cells in CB, and therefore children who underwent UCBT did not use ATG during pretreatment, led to differences in the use of ATG during the conditioning regimen in the two groups of children. Consequently, differences in graft type between the WAS and CGD groups of children in this study resulted in differences in graft MNC content and CD34+ cell count as well as conditioning regimen. In the present paper, we focused on the effect of two variables (transplantation age and graft type) on immune recovery in those with WAS and CGD.

In this paper, the median age of children who received transplants in the WAS group was significantly younger than that of children in the CGD group, so does the age at transplantation contribute to the difference in immune reconstitution between the two groups of children? Patient age at transplantation has been recognized as a prime determinant of the speed and quality of immune reconstitution since the start of this research (12). It was shown that patients younger than 8 years who underwent allo-HSCT exhibited higher absolute lymphocyte counts at Day 30 posttransplantation and better CD4+ T-cell recovery at Day 100 posttransplantation (13). Another previous study reported that younger recipient age was associated with higher total and memory B-cell counts after transplantation, pointing to a potential role of nonhematopoietic/microenvironmental factors (14). We divided the WAS and CGD groups into different age groups (<1 year, 1-3 years, 3-5 years, >5 years); selected the age group with the largest number of children, i.e., children who underwent transplantation at 1-3 years; and compared their posttransplantation lymphocyte subpopulation reconstitution (**Table 3**). The results showed a higher level of lymphocyte subpopulation reconstitution in children who underwent transplants at 1-3 years in the WAS group compared to children who underwent transplants at 1-3 years in the CGD group. The finding was generally consistent with the findings of immune reconstitution in children of all ages in the WAS and CGD groups. Considering the effect of graft type, a comparison was also made between the graft types of children in the age group 1-3 years (**Table 4**), and it was observed that there was no significant difference between the graft types of children in the WAS and CGD groups within this age group. Therefore, in children with WAS and CGD who received allo-HSCT at the age of 1-3 years, we have not found an effect of age on immune reconstitution in both groups.

We next investigated the effect of graft type on immune reconstitution in WAS and CGD groups. As more patients in the WAS group underwent UCBT, we divided the patients in the WAS group into two groups according to UCBT and non-UCBT and compared the differences in lymphocyte subpopulation levels between the two groups after transplantation (**Table 5**). We found children who underwent non-UCBT had higher B-cell counts than children who underwent UCBT in the WAS group at Days 15 and 30 after allo-HSCT, but children who underwent UCBT had higher B-cell counts than children who underwent non-UCBT in the WAS group on Days 100 and 180 after allo-HSCT. This phenomenon seems to indicate that CB has strong B-cell reconstitution potential after allo-HSCT. Previous studies suggested that B-cell recovery after UCBT was faster than after sibling peripheral blood transplantation because of better B-lymphocyte reconstitution *in vitro* and *in vivo* in CB containing a higher proportion of progenitor cells (15–18). Several articles report an advantage of CB over bone marrow (BM) or peripheral blood stem cells in terms of B-cell recovery time and B-cell differentiation (15, 19, 20). A previous study concluded that the total number of B cells, non-transformed memory cells, and transformed memory B cells was higher in the CB compared to the BM or peripheral blood (15). Children with WAS have a strong B-cell reconstitution potential after UCBT, which can also be explained by the higher number of B lymphocyte progenitor cells in CB compared to BM (16). Reconstruction of CD4+ T cells in recipients receiving different graft sources varies considerably (19, 21). Early CD4+ T-cell reconstitution is significantly affected by the components of the conditioning regimen and is extremely late when T-cell depletion (e.g., ATG) is used in the conditioning regimen (22). An investigation noted that the non-use of ATG in conditioning regimens in patients with UCBT was associated with rapid CD4+ T-cell reconstitution (23). In this study, children underwent UCBT in the WAS group had higher CD4+ T-cell counts than children underwent non-UCBT, considering that none of the children underwent UCBT in the WAS group used ATG in the conditioning regimen (0%, 0/24), whereas children underwent non-UCBT in the WAS group partially used ATG in the conditioning regimen (63%, 29/46). Therefore, we considered that the T-cell counts of children with UCBT in the WAS group in this study were significantly higher than those of children with non-UCBT, which may be highly associated with the use of ATG. A higher proportion of children in the WAS group received UCBT compared to the CGD group; thus, UCBT may be partly responsible for the faster immune reconstitution in children with WAS after transplantation than in children with CGD.

In addition to the effect of UCBT, we additionally did a comparison of the counts of lymphocyte subpopulations in non-cord-blood-transplanted children in the WAS and CGD groups (**Table 6**). Coincidentally, lymphocyte counts were also higher in the non-cord-blood-transplanted children in the WAS group compared to the non-cord-blood-transplanted children in the CGD group. Therefore, UCBT cannot fully explain the faster immune reconstitution in children in the WAS group than in children in the CGD group. Previously, we found that the effect of age at transplantation on immune reconstitution was relatively small in both groups, and that children with non-UCBT in the WAS group still had a faster rate of immune reconstitution than children with non-UCBT in the CGD group when the effect of UCBT was not considered, therefore, we hypothesize that there may be an effect of the primary disease itself in addition to the effect of CB.

Immunoglobulins and peptides were used as a crude proxy for B-cell counts and function, and the present study also analyzed IgM, IgA, IgG, C3, and C4 serum levels after allo-HSCT in WAS and CGD groups of children (**Table 7**). Past studies have shown that immunoglobulin levels seem to recover simultaneously with B-cell reconstitution, where the recovery of Ig subclasses usually occurs in a unique order (15, 24). In this article, kids with CGD had higher IgA values than those with WAS at day 360 posttransplantation. However, B-cell counts were higher in children with WAS than in those with CGD at days 100 and 180 post-transplantation, with no statistical difference between the two groups at day 360 post-transplantation, indicating that B-cell reconstitution levels were inconsistent with immunoglobulin recovery in children in the WAS and CGD groups. This may be because the number of B cells is not synchronized with the recovery of secretory function, but this speculation needs to be confirmed by further studies. The results of this study also showed that children with CGD had higher C3 values than those with WAS at day 100 posttransplantation, and children with CGD had higher C4 values than those with WAS at day 360 posttransplantation. Complement is one of the important mechanisms by which antigenic antibodies exert their immune effects. In children with CGD, where the number of B cells recovered is low, more complement is beneficial to balance the immune deficiency caused by insufficient number of B cells, so the higher level of C3 and C4 in children with CGD may be a feedback effect from insufficient number of B cells. Regarding IgG reconstitution, most children in our center received regular gammaglobulin infusions during the first months posttransplantation, which may have led to similar levels of IgG reconstitution in the both groups of children.

In the above analysis, it was demonstrated that the difference in age of transplantation had a relatively small effect on immune reconstitution in children aged 1-3 years, whereas differences in graft type may have a partial effect on immune reconstitution in children with WAS and CGD, and in addition, we speculate that it was associated with the fact that WAS and CGD are two different primary diseases.

We also analyzed the posttransplantation infections in children with WAS and CGD in different age groups (<1 year, 1-3 years, 3-5 years, >5 years) (**Table 8**), and results showed that among children under 1 year old who underwent transplantation, the CGD group had a higher number of posttransplantation EBV infections and fungal infections compared to the WAS group. Among children 1-3 years old who underwent transplantation, the CGD group had a higher number of posttransplantation EBV infections and CMV infections than the WAS group. The number of posttransplantation fungal infections was also higher in the CGD group than in the WAS group among children over 5 years of age who received transplants. When comparing the pre and post-transplantation infections in the two groups, we found that before transplantation, the number of CMV infections in children with WAS was higher than that in children with CGD, while the number of EBV and fungal infections was lower than that in children with CGD. After transplantation, the number of CMV, EBV and fungal infections was significantly lower in children with WAS than in those with CGD. This phenomenon suggests that faster immune reconstitution in children with WAS may reduce the incidence of fungal and viral infections after transplantation.

In addition to analyzing the impact of differences in immune reconstitution on posttransplantion infection in the two groups of children, we also evaluated the impact of immune reconstitution on the duration of posttransplantion immunoglobulin infusion (**Table 1**). The analysis showed that the time to final immunoglobulin infusion was significantly less in children in the WAS group than in those in the CGD group after transplantation. This result confirms that the faster immune reconstitution in children with WAS may shorten the time to immunoglobulin infusion in children with WAS. The Chinese Expert Consensus on Vaccination for Children in Special Health Status recommends that after 1 year posttransplantation, immune function is normal and all types of inactivated vaccines can be administered (25). Children are in a special immune state after transplantation, and it takes 1-2 years for the immune function to be reestablished; therefore, most children in our center are not vaccinated within one year after transplantation.

Overall, this study pioneered a study on the differences in immune reconstitution posttransplantation in patients with WAS and CGD. We found that immune reconstitution is faster in children with WAS than in children with CGD, and this difference may be related to the fact that the two diseases are different primary diseases and to graft types. Our retrospective study has limitations regarding the irregularity, sample size, and patient follow-up time of immunosurveillance *via* flow cytometry or immunoturbidimetry after transplantation. In the future, we need to keep applying the existing standardized immune reconstitution monitoring methods and explore more patterns for immune reconstitution in children with different primary diseases undergoing allo-HSCT, which can guide physicians in using antimicrobial drugs and screening for pathogens and the development of strategies to enhance immune reconstitution, which will significantly improve the efficacy of allo-HSCT in children with immunodeficiency diseases.

## Data availability statement

The raw data supporting the conclusions of this article will be made available by the authors, without undue reservation.

## Ethics statement

The studies involving human participants were reviewed and approved by the Children’s Hospital of Chongqing Medical University Research Ethics Committee. Written informed consent to participate in this study was provided by the participants’ legal guardian/next of kin.

## Author contributions

All authors contributed to the study conception and design. Data collection were performed by YZ and LJ. Data analysis were performed by YZ. The first draft of the manuscript was written by YZ and all authors commented on previous versions of the manuscript. All authors read and approved the final manuscript.
